# Post-infectious headache: a reactive headache?

**DOI:** 10.1007/s10194-011-0346-0

**Published:** 2011-05-05

**Authors:** Sanjay Prakash, Niyati Patel, Purva Golwala, Rushad Patell

**Affiliations:** 1 Department of Neurology, Medical College, SSG Hospital, O-19, Doctor’s Quarter, Jail Road, Baroda, 390001 Gujarat India; 2 Department of Medicine, Medical College, Baroda, 390001 Gujarat India

**Keywords:** Headache, Chronic daily headache, New daily persistent headache, Secondary headache, Post-infectious, Infection

## Abstract

Post-infectious disease syndrome includes both neurological and non-neurological disorders. However, headache as an isolated or a presenting complaint of post-infectious illness has not been well acknowledged in the literature. In this retrospective observation, patients having daily headache of more than 1 week and <4 weeks duration were included. We divided this group into patients having headache with preceding history of febrile illness in the recent past and patients without such history of febrile illness. We compared clinical features and therapeutic responses of various drugs between the groups. There were no significant differences in demographic features in these groups. However, associated neck pain, nausea, photophobia and meningeal signs were more prevalent in patients having history of preceding infection. A relatively lower proportion of subjects showed complete response to drugs at 3 months in post-infectious group. Good responses were noted to steroids in post-infectious group. In conclusion, a subset of patients with daily headache may be because of post-infectious pathology and treatment in the early stage may prevent it from becoming chronic. Large prospective studies are required to confirm these observations.

## Introduction

Headaches attributed to infectious causes are important secondary causes of headache. The ICHD-II divides “headache attributed to infection” into four categories: (1) headache attributed to intracranial infection, (2) headache attributed to systemic infection, (3) headache attributed to HIV/AIDS, and (4) chronic post-infection headache [1].

A diagnosis of headache attributed to an infection usually becomes definite only when the headache resolves or greatly improves after effective treatment or with spontaneous remission of the infection. When the intracranial infection is effectively treated or when it remits spontaneously, but headache persists for more than 3 months, the diagnosis of chronic post-infection headache is made. There is only one subgroup of chronic post-infection headache, chronic post-bacterial meningitis headache. In this group, the headache is a direct continuation of headache attributed to bacterial meningitis [2]. Viral meningitis (aseptic meningitis) may also cause chronic post-infection headache [2], and ICHD-II acknowledged this as post-non-bacterial infection headache in the Appendix (A9.4.2) [1]. Besides these, infectious etiology has been suggested in a subset of patients with NDPH, where infection is considered as a triggering factor for generation of headache [3].

ICHD-II describes (chronic) post-infection headache as a direct continuation of headache attributed to bacterial meningitis. However, post-infectious disorder is usually described when symptoms and signs develop following recovery from an infection elsewhere in the body [4]. Various post-infectious neurological and non neurological disorders [acute disseminated encephalomyelitis (ADEM), chronic fatigue syndrome] may have headache as one of the accompanying symptoms. However, headache as an isolated or a presenting complaint of post-infectious illness has not been well acknowledged in the literature. Recently, a case series on post-infectious variant of NDPH has been reported in which all nine patients had a history of febrile illness before the onset of headache [5]. To look for the spectrum of post-infectious headache, herein, we retrospectively studied the headache profiles where patients reported history of febrile illness before the development of headache.

## Materials and methods

This study was conducted as a retrospective chart review of patients seen in neurology department in our institute from September 2008 to August 2010. The study did not require approval by the local ethics committee as per the local regulations for retrospective observations. The patients having daily or near daily headache for more than 1 week and <4 weeks duration, and with minimum follow-up of 3 months duration were included in the study. A few patients were interviewed on telephone to complete the follow-up. These patients were specifically reviewed for the presence of febrile illness in the preceding 4 weeks before the onset of headache. We divided the patients into two broad groups: patients having headache with preceding history of febrile illness in the recent past and patients without such history of febrile illness. We compared clinical features and therapeutic responses of various drugs between the groups.

The headache duration of more than 1 week was included to just rule out the possibility of an episode of tension headache according to ICHD-2 criteria. The headache duration of more than 4 weeks was excluded to minimize recall bias for the presence of preceding illness. The patients having headache since the onset of febrile illness were excluded from the observation to rule out the possibility of headache attributed to systemic infection, chronic post-bacterial or non-bacterial infection headache (ICHD-2: 9.2, 9.4, and A9.4.2). All these three headache groups have headache with evidence of infections. Patients having past history of headache in the last 1 year were also excluded from the observation. The patients having history suggestive of red flags (as suggested by Goadsby and Raskin [6]) were excluded from the observations. The patients having abnormal neurological examinations (except for the presence of meningeal signs) were excluded.

The majority of patients were seen and examined by a neurologist and further investigational studies, especially neuroimaging and lumbar puncture were requested when clinically indicated to exclude secondary etiologies. Management decisions were made at the discretion of the treating physicians.

## Results

Thirty-four patients having daily or near daily headache of more than 1 week and <4 weeks duration were identified. We classified these patients in two broad groups: patients with and without history of febrile illness in last 4 weeks. Twenty-three patients (68%) had a preceding febrile illness before the onset of headache. Epidemiological and clinical features of both groups were compared (Table 1). There were no significant differences in age, sex, and duration of headache between the groups at the time of presentation. The site of pain was also comparable in both the groups. None of the differences were statistically significant. However, associated neck pain was more in group having history of infection (61 vs. 36%). The quality of pain was predominantly dull or pressing type, in both the groups in about 75%. Most of the patients in both the groups had mild to moderate intense headache. However, continuous pain with or without fluctuation was more common in post-infectious headache (78 vs. 64%). Nausea, photophobia and meningeal signs (neck rigidity, Kernig’s sign) were more prevalent in patients having history of febrile illness. All patients had headache on nearly daily basis at the time of presentation. Fourteen patients (61%) in post-infectious group and five patients (46%) in non-infectious group had daily headache since the onset of pain. Headache became continuous within 3 days of the onset in another five patients (22%) of post-infectious group and two patients (18%) of second group. Continuous head pain with not much or minimal fluctuation was the most common type of headache pattern.

**Table 1:** Differences between headache preceded by infection and headache with no prior history of recent infection

Parameters	Headache preceded by infection (*n* = 23)	Headache with no prior history of recent infection (*n* = 11)
Age (years) (mean, range)	32.5 ± 12.6, 17–55	34.8 ± 14.5
Sex (F:M)	12:11	6:05
Duration of headache (weeks)		
1–2	10 (44%)	4 (36%)
2–3	7 (30%)	4 (36%)
3–4	6 (26%)	3 (27%)
Location of pain		
Holocephalic	10 (44%)	5 (46%)
Parieto occipital	8 (35%)	3 (27%)
Fronto temporal	5 (22%)	3 (27%)
With neck pain	14 (61%)	4 (36%)
Quality of pain		
Pressing	17 (74%)	8 (73%)
Throbbing	6 (26%)	3 (27%)
Variability of headache		
Intermittent daily pain	5 (22%)	4 (36%)
Constant with no or little fluctuations	14 (61%)	6 (55%)
Constant with frequent exacerbations	4 (17%)	1 (9%)
Severity		
Mild to moderate	21 (91%)	9 (82%)
Severe	2 (9%)	2 (18%)
Associated symptoms and signs		
Nausea	7 (30%)	2 (18%)
Photophobia	7 (30%)	2 (18%)
Phonophobia	2 (9%)	1 (9%)
Cranial autonomic feature	1 (4%)	0
Meningeal sign	9 (39%)	3 (27%)
Body pain	5 (22%)	2 (18%)
Neuroimaging		
Normal	13/13	4/4
CSF examinations		
Pleocytosis	3/9	0/3
Provisional diagnosis (at the time of presentation)		
Probable migraine	8	4
Probable CTTH	11	6
Probable NDPH	4	1
Treatment		
Dothiepin	12	6
Amitriptyline	4	5
Valproate	7	4
Propranolol	4	0
Leviteracetam	3	0
Paracetamol	8	5
Naproxen	15	6
Indomethacin	5	1
Steroids	10	2
Follow-up and treatment response		
At 3 months		
Complete response	8 (35%)	6 (55%)
Marked response with episodic headaches (1–5 attacks/week)	10 (43%)	4 (36%)
No or minimal response	5 (22%)	1 (9%)
Response to steroids (at 3 months)	(10 patients)	(2 patients)
Marked and immediate	4/10 (40%)	0/2
Moderate response	3/10 (30%)	1/2
Mild to no response	3/10 (30%)	1/2
Response to steroids (IV MPS) (at 9 months)	(7 patients)	(2 patients)
Marked and immediate	3/7 (42%)	0/2
Moderate response	2/7 (29%)	1/2
Mild to no response	2/7 (29%)	1/2

The details of preceding febrile illness and its relation with headache have been summarized in Table 2. Only five patients received definitive diagnoses during their febrile illness (dengue 2 patients, H1N1-2 patients, salmonella typhi 1 patient). Other 18 patients had non-specific infections. Most of them had upper respiratory tract infections (rhinitis 8, pharyngitis 7). Three patients had a history suggestive of gastrointestinal infections. In most of the patients (21 out of 23), headache started within 3 weeks of the cessation of febrile illness.

**Table 2:** Relation of infection with headache

Parameters	Headache preceded by infection (*n* = 23)
Type of illness	
Specific illness	
Dengue	2
H1N1	2
*Salmonella typhi*	1
Non specific	
Rhinitis	8
Pharyngitis	7
Gastroenteritis	3
Time interval with headache onset (weeks)	
<1	8
1–2	7
2–3	6
3–4	2

Provisional diagnoses, at the time of presentation, were either probable chronic migraine or probable CTTH or probable NDPH in all the patients.

Neuroimaging was essentially normal or was not casually related in both the groups. CSF examinations were performed in all patients of both groups having meningeal signs. CSF pleocytosis (>5 cells/mm) were noted in three patients in post-infectious group (out of 9 in whom CSF analysis was done) and in one patient (out of 3 patients) in other group. CSF protein and glucose were within normal limit.

All patients received medications. The drugs used and treatment responses are summarized in Table 1. All patients have follow-up of minimum 3 months durations (as inclusion criteria). The treatment response at 3 months of the onset of symptoms was analyzed.

A relatively lower proportion of subjects showed complete response at 3 months in post-infectious group as compared to non infectious group (35 vs. 55%). Five patients (22%) in post-infectious group and 1 patient (9%) in other group showed minimal or no response at the end of 3 months of symptom onset, and now these patients fulfilled the criteria of NDPH. Other patients, 10 patients (43%) in post-infectious group and 4 (36%) in non-infectious group, had mild to moderate improvement in headache profiles.

Steroids were supplemented along with other drugs in a few patients who did not show response to usual therapies. None of the patients received steroids at the beginning of the therapy. Oral steroids were added after 2–6 weeks of usual therapies. 10 patients of post-infectious group and 2 in other group received steroids (mainly oral prednisolone, 1 mg/kg body wt) in the first 3 months. Steroids were given for 7 days to 2 weeks duration; four patients (40%) in post-infectious group had a marked immediate effect (within 2–4 days) to steroid. Another three patients showed moderate effect to steroid. One patient (out of 2) in non-infectious group showed moderate effect to prednisolone.

Fifteen patients (65%) in post-infectious group and five patients (45%) in other group still had headache at the end of 3 months. We were able to get follow-up of a few more patients in both the groups (9 patients in first group and 2 patients in second group) at the end of 9 months. Three patients in post-infectious group and both patients of non infectious group were almost symptom free. Another four patients had marked improvement (1–4 attacks/month). However, two patients in the post-infectious group still had continuous headache (although with mild back ground pain with reduced frequency of exacerbations).

Three of these nine post-infectious group patients had received oral steroid during first 3 months and had noted steroids as effective drug, at least moderately effective for a few weeks. These three patients and four more patients received a pulse therapy of IV MPS for 3–5 days. The response to steroids in these seven patients of post-infectious group were: marked immediate effect (3 patients), moderately effective (2 patients), and minimal response (2 patients). One patient of the non-infectious group also received IV MPS and this patient showed moderate response to steroid.

## Discussion

In this retrospective observation of continuous or near daily headache of shorter duration (1–4 weeks), we specifically looked for the presence of history of febrile illness in the recent past (<4 weeks). Post-infectious disorders usually start after an active infection [4]. The distinction of infectious from post-infectious process is important as infectious process depends on the continued presence of viable microorganisms and should be treated with antimicrobial therapy, while post-infectious process ought to be blocked by inhibition of the involved host process [4].

We compared patients with continuous headache of shorter duration with history of recent infection in the past to patients with similar type of headache, but without any recent febrile illness. The demographic features of both groups were substantially similar. Although there were no statistically significant differences between the groups, a few striking differences between the groups were noted. The post-infectious group was more likely to have associated neck pain, body pain, nausea, photophobia, and meningeal signs. The post-infectious group had more continuous headache.

None of the patients fulfilled criteria of any primary headache disorders. The patients having headache since the onset of febrile illness were excluded from the observation to rule out the possibility of headache attributed to intracranial infections, headache attributed to systemic Infection, chronic post-bacterial or non-bacterial infection headache, and aseptic meningitis (ICHD-2: 9.1, 9.2, 9.4, A9.4.2 and 7.3.2). However, due to the presence of neck pain, meningeal signs and pleocytosis, possibility of these differential diagnoses cannot be ruled out completely, especially headache attributed to lymphocytic meningitis (9.1.2) and headache attributed to systemic infection (9.2). However, lymphocytic meningitis because of direct involvement of microorganisms seem to be less likely as patients did not have fever (with headache) and general feeling of illness. Headache attributed to lymphocytic meningitis usually resolves within 1 week (notes of 9.1.2). In our case series, all patients had headache duration of more than 1 week (as inclusion criteria). In headache attributed to systemic infection, headache develops during the systemic infection and resolves within 72 h after effective treatment of the infections. None of the patients had these features. Another possibility in our patients is headache attributed to aseptic (non-infectious) meningitis (7.3.2). IHS recognizes ibuprofen, penicillin, trimethoprim and others as causative factors for the generation of headache attributed to aseptic (non-infectious) meningitis. Few of our patients received these drugs in their febrile episodes. However, possibility of this headache disorder is less likely as patient had already left these drugs well before the onset of headache.

The diagnosis of headaches is mostly clinical, according to the criteria of the International Headache Society (IHS) and such strict criteria are important for scientific reasons (research purpose), but, in clinical practice, they seem to be too restrictive, and we find a large number of patients not fitting in any criteria. This problem is more in patients with chronic daily headache (CDH). The literature is silent on classifying sub-acute onset daily headaches. Sudden onset daily headache is a characteristic of NDPH. Our most patients had headache duration of 1–2 weeks (at the time of presentation); 1–2 weeks duration was not sufficient enough to label this type of headache as NDPH. An episode of headache may present up to 7 days in tension-type headache (according to the IHS criteria). The prevalence of TTH is far common in comparison to NDPH and other headache disorders. Therefore, probable CTTH was the most common provisional diagnosis in both groups. Probable chronic migraine and probable NDPH were the other two (differential) diagnoses. The treatment response at the 3 months was less favorable in post-infectious group. 65% patients in post-infectious group and 45% in other group still had continuous or intermittent headache at the 3 months and most of them fulfilled the criteria of CDH.

Neck pain is a common occurrence in patients with CDH and it has been associated with poor outcome [7]. The co-occurrence of headache and neck pain may be because of common pathogenesis, a causal association, or a common confounding factor. However, the presence of meningeal signs and pleocytosis in CSF in a few patients indicate involvement of meninges, and both headache and neck pain may be because of common pathogenesis (meningeal involvement).

The presence of meningeal signs in patients with recurrent or chronic headache has not been well explored in the literature. We noted only one article in the literature where signs of meningeal irritation were observed in patients with CDH [8]. This observation was done in children and adolescents, in which 97% children with tension headache and 12% children with migraine showed meningismus and the authors suggested that tension-type headache is basically a secondary headache disorder and its main clinical features is because of meningismus. The authors suggested a possibility of mild sterile (possibly autoimmune) inflammation of meninges because of preceding infection and minor trauma to head and/or back [8]. Meningeal sign in the form of neck stiffness (with nausea, photophobia, phonophobia) was a common accompanying features (50%) in Li and Rozen’s [9] case series of NDPH.

Post-infectious disease syndromes are immune-mediated inflammatory diseases that include both neurological and non-neurological disorders. However, headache as an isolated or a presenting complaint of post-infectious illness has not been well acknowledged in the literature. Recently, a case series of post-infectious NDPH has been reported where the onset of headache was preceded by an episode of febrile illness [5]. Besides it, NDPH may start during a febrile episode and may present for many years or even life long. In Mack series of NDPH, 43% patients had onset of their symptoms during an infection [3]. In Li and Rozen’s [9] case series NDPH onset occurred in relation to an infection or flu-like illness in 30% cases. Serological evidence of present or past viral infections has also been demonstrated in high proportion in patients with NDPH [10, 11].

Migraine can have an abrupt transition from episodic to chronic form (transition may be abrupt, like onset of NDPH). Mack [3] reported that such abrupt transition (in children) is most commonly associated with febrile illness. A few other authors have also demonstrated flu-like illness/sinusitis as risk factors for abrupt transition from episodic to chronic daily headache disorders [12]. The exact pathophysiology of abrupt onset of NDPH and abrupt transition of episodic headache to chronic daily headache has not been explored. However, a close temporal relation with febrile illness may suggest a possibility of post-infectious process in daily or near daily headache disorders.

Favorable response to immunotherapy is one of the indirect evidences to consider a disease as a post-infectious disorder [4]. Most post-infectious disorders show a response to steroids and other immunosuppressive drugs. The literature on the use of steroids in the treatment of headache disorders is conflicting. Most studies are done on migraine patients. The available data suggests that steroids may be more useful in subjects with migraine lasting longer than 72 h [13]. A few other persistent headache disorders have also shown response to various steroids. The case series on post-infectious NDPH of shorter duration (a few of them had headache duration of <3 months at the time of observation) have also shown response to intravenous steroids [5].

Most of our patients who received steroid showed moderate to marked response on headache. Although headache management and treatment were not standardized in the patients, a response to steroids may be indicative of immunological/inflammatory mechanisms for the generation of headaches.

Evidence of infectious (or post-infectious) etiology associated with a few diseases has been supported by winter and spring peaks in presentation. There were limited studies on the seasonal variation of headache disorders. However, available data indicate increased frequency of headache attacks in autumn–winter and least attacks in summer [14, 15]. In one of the largest case series of NDPH, September was the most common month of onset [16]. This headache profile matches with the seasonal variations of infections, especially respiratory infection. Viral infection is a common occurrence in the general population. A latency period of <30 days has been suggested as a possible link between a febrile event and a post-infectious entity [17]. All our cases have <30 days of latency. Therefore, a possibility of causal link exists in our patients.

### Pathogenesis

It is difficult to speculate on the mechanisms of post-infectious headache, as current knowledge of the pathophysiology of even classical primary headache disorders is limited. Acute onset of headache in our patients and in patients with NDPH is highly suggestive of some etiology. Abrupt transition of episodic migraine to chronic migraine also suggests similar possibilities. Vanast [18], in 1987, suggested autoimmune disorder with a persistent viral trigger for chronic benign daily headache (NDPH). Tissue specificity is a general feature of post-infectious immune-mediated conditions [4]. Our case series and review of the literature, especially Almazov and Brand [8] observations, and Li and Rozen’s [9] case series, suggest that meningeal involvement may the primary site for the generation of post-infectious headache. Rozen-Swidan [19] demonstrated high-proinflammatory cytokine (TNF-α) in CSF in patients with NDPH and treatment refractory chronic migraine. Serum proinflammatory cytokine level was normal and the authors suggested CNS activation in these patients.

Meninges are a type of connective tissue membranes. Another important connective tissue membrane in the body is synovial membrane of the joints. Reactive arthritis (ReA) is a post-infectious disease entity of synovium/joints. We speculate that mechanisms responsible for the generation of joint pain in ReA may be primary mechanisms for the generation of headache in our patients and probably in a subset of patients with NDPH. The course of ReA is comparable to that of NDPH: short or self-limiting form, continuous, and remitting form [20]. Although acute ReA may be associated with low TNF-α, chronic ReA shows high production of TNF-α (like NDPH) [21]. ReA is the persistence of pathogenic organisms or its products in the joint/synovium leading to local immune response. We speculate a local immune reaction in the meninges for the generation of headache in our patients and a subset of NDPH. Therefore, such headache could be termed as ‘Reactive Headache’. 

The meninges are most often thought of as simply a protective barrier between the CNS and the periphery. However, recent observations suggest that cellular composition of meninges include a large number of immune cells (such as fibroblasts, macrophages, dendritic cells, and mast cell) and each of which are capable of releasing proinflammatory, neuroexcitatory mediators in response to inflammatory/immunological challenges and meninges may have a potential role in modulation or generation of pain, as it is richly innervated by trigeminal nerves [22, 23]. The meninges have also been recently implicated in pain processing even at spinal levels [22]. Recently, Wieseler et al. [23] have shown facial allodynia following supradural administration of both the inflammatory soup (bradykinin, histamine, serotonin, and prostaglandin E2) and the immunogenic stimulus (gp120) in awake and freely moving rats. These observations were associated with meningeal inflammation and activation of meningeal resident immune cells suggesting a role of immunological mechanisms in induction of meninges-induced pain. The high CSF proinflammatory cytokine (TNF-α) in Rozen–Swidan observations [19] may be, at least partly, because of meningeal involvement.

Our case series and review of the literature suggest that headache may occur in temporal relation of a febrile illness. We included only those patients who had symptoms free period between febrile illness and the development of headache to differentiate such type of headache with headache directly related to febrile illness. However, any post-infectious illness may develop within 2 days after an antigenic challenge and post-infectious process may start within febrile periods. As noted above, flu-like illness or other febrile illnesses are the risk factors for the development of NDPH and acute transition of episodic headaches into chronic daily headache. We speculate that a subset of patients in these groups may be because of post-infectious mechanisms. We further speculate that if an infection (or post-infectious process) can trigger chronic headaches (>3 months duration), there is a possibility to develop NDPH-like headache (or daily headaches) for shorter duration (sub-acute NDPH) with variable prognosis (some as self-limiting condition and some turning into refractory NDPH after 3 months).

Most post-infectious illness have better prognosis if treated earlier [4]. Even the case series of post-infectious NDPH of shorter duration have shown favorable response to IV MPS [5]. Therefore, identification of this group is important as early interventions may prevent them from being refractory, as chronic daily headaches have poor prognosis. Besides it, a few percentage patients (up to one-third) of most of the post-infectious diseases do not have clinically evident antecedent infections during the prior few weeks [4].

Taken together, our case series, association of infection as an important factor for the generation of CHD, and our speculation on possible pathogenesis for this association suggest that a subset of patients with daily or near daily headache may be because of post-infectious immune pathology. Our observations suggest that a few of them may not respond to usual therapies of headache and may turn into CDH (>3 months), and early immunosuppressive drugs (steroids) may be helpful in this group of patients. Therefore, it is important to look for the potential cases of NDPH and other CDH in early stage. As our observation is retrospective, it must be substituted by future prospective studies. We suggest prospective studies on patients with daily or near daily headache of sub-acute onset for the risk factors for the chronification of headache with special reference to post-infectious etiology (both clinical and laboratory features). Epidemiological studies on headache prevalence/incidence especially during outbreaks of various infections, such as respiratory, gastrointestinal or other infections may also be done to confirm this association. If we can identify microbial agents that trigger an autoimmune disease, it may be possible to treat and even prevent the disease.

### Limitations of our observations

It is a retrospective study and possibilities of unrecognized selection bias and recall bias exist. Our observation did not have large enough numbers to uncover true differences in the two groups. All our patients gave history of antecedent infections before the onset of headache. However, confirmed laboratory diagnosis was available in only five patients. We do not have any serological evidence of infections in most of the patients. In addition, headache management and treatment were not standardized. Besides these, we cannot rule out even the possibility of other cause of headache (secondary), as full evaluation for secondary headache was not done. Neuroimaging was not done in all the patients. A few secondary headache disorders may resolve even without treatment. Besides these, our observations cannot be generalized as our sample of patients may not truly represent patients with new onset headaches due to referral and other biases. Moreover, geographical variation of infectious pathogens should also be taken into consideration for such type of observations.
